# Refocusing vector assessment towards the elimination of onchocerciasis from Africa: a review of the current status in selected countries

**DOI:** 10.1093/inthealth/ihx066

**Published:** 2018-02-19

**Authors:** Daniel Boakye, Jamie Tallant, Aime Adjami, Samfo Moussa, Afework Tekle, Magda Robalo, Maria Rebollo, Pauline Mwinza, Laston Sitima, Paul Cantey, Charles Mackenzie

**Affiliations:** 1 WHO/AFRO/ESPEN Laboratory, Ouagadougou, Burkina Faso; 2 The END Fund, New York, USA; 3 WHO/AFRO/ESPEN, Brazzaville, Congo; 4 WHO, Geneva, Switzerland; 5 Onchocerciasis Control Programme, Malawi Ministry of Health, Malawi; 6 The Taskforce for Global Health, Atlanta, GA, USA

**Keywords:** Assessment, Challenges, Cross-border, Onchocerciasis, *Simulium*, Vector

## Abstract

Measures to control onchocerciasis have been in place for well over 30 years. Recently, programs have turned from disease control towards transmission elimination. The absence of infective larvae in the black fly *Simulium* sp. vector is central to defining elimination, and assessments of infectivity by O150 polymerase chain reaction in the vector not only provide valuable information to programs, but are also required for verification of elimination. The status of transmission in black flies was assessed in five countries in the African region during 2014 and 2015. Several of these countries were evaluated because of promising results from epidemiological studies in humans. No infective flies were found in two countries. Infective flies were found in the other three, despite the absence of infection in humans (as evaluated by skin-snip microscopy). Ongoing transmission as demonstrated in the black flies could be due to a variety of factors, including lack of treatment of hypo-endemic areas and cross-border issues. Challenges identified during the course of the entomological work suggest that there is a need for improved selection of vector collection sites and vector collection periods in order to improve fly catches. Two important challenges to achieving elimination identified were definition of the hypo-endemic zones and establishing the existence of areas of cross-border transmission occurring between countries.

## Introduction

Human onchocerciasis is transmitted through the bite of the infected *Simulium* black fly vector. Onchocerciasis is a disfiguring and economically detrimental disease that causes skin and eye disease. Infection in the black fly is a direct indicator of the presence of transmission, and the demonstration of complete breaking of transmission requires demonstration of a lack of infective black flies by O150 polymerase chain reaction (PCR). Fly evaluations have to be completed in addition to human serological evaluations, in order to satisfy WHO criteria for stopping mass drug administration and verification of elimination of human onchocerciasis.^1^

Attempts to break transmission by eliminating the vector were the first internationally supported action aimed at controlling the blindness and debilitating skin disease caused by the parasite that the vector transmits. Larvacidal spraying of the riverine vector breeding sites was used in West Africa in the early 1970s, but failed to completely eliminate the transmission of disease in these areas, although there were major reductions in disease and focal areas of elimination.^2^ The introduction of the chemotherapeutic agent, ivermectin, donated by MSD, also known as Merck & Co., Inc., Kenilworth, NJ, USA, in 1987, and its use over the past 30 years has significantly reduced clinical disease.^3^ This success has catalyzed the global effort against this disease to focus on a goal of elimination.^4,5^ Indeed, the elimination of transmission has been demonstrated in some African onchocerciasis foci, where assessments in humans and the vector have shown that transmission has ceased.^6,7^

The elimination of onchocerciasis in Africa was not felt to be feasible until relatively recently.^8^ However, after some successes in the Americas, interest grew in Africa and work began to determine if the disease could be eliminated in Africa as well. The African Programme for Onchocerciasis Control (APOC) began the transition to elimination of transmission after reports of successful elimination in Uganda,^6^ supporting studies in Mali and Senegal,^4^ in Bioko Island in Equatorial Guinea^9,10^ and now from Sudan.^11^ This change in goal was built into the objectives of the project that replaced the APOC after its closure in 2015, the Expanded Special Project for the Elimination of NTDs (ESPEN).

As the APOC began to focus on achieving elimination from the continent, it became clear that an effort to reassess the current status of the infection in each country was needed. The reassessment would, in addition to providing important information about program impact, be an opportunity to test and refine the specific methodologies needed to document interruption of transmission. The APOC and partners sponsored the review of the available epidemiological data to pick areas for entomological evaluation. This report provides some of the details of those evaluations and identifies some of the challenges identified by them.

## Methodology

### Overall approach

The overall goal of the study was to understand the capabilities of selected countries to carry out field assessments for entomology and identify any major challenges that need to be addressed. Two major activities were undertaken:

- a historical review of existing country data regarding transmission was performed;
- entomological assessments in five countries were implemented in 2014–2016.

The five countries that were selected for entomological evaluations were chosen because of historical information that suggested that the country had reduced (or possibly interrupted) transmission.

### Historical review

Existing reports and publications from APOC were examined for information regarding transmission. Histories of the five countries (Niger, Senegal, Malawi, Chad and Guinea Bissau) were examined in detail for information related to control strategies (e.g. ivermectin mass drug administration [MDA] and vector control activities) used by the country and data regarding the status of transmission (e.g. reported MDA coverage, results of skin-snip epidemiological surveys). Countries were then supported for entomological evaluations in 2014 and 2015. Previous skin-snip surveys in four of the countries performed under APOC supervision suggested that transmission was at extremely low levels; no infection was detected in skin-snip surveys in several sites.^12^ Niger was included as reports from the country program indicated that infection and transmission levels in this country were also likely to be extremely low.

### Entomological methods

Entomological samples collected in 2014–2015 from the selected countries were processed in the ESPEN Onchocerciasis Laboratory in Ouagadougou, Burkina Faso (formerly the Multi-Disease Surveillance Centre Laboratory). The entomological methods used were the current standard approaches used to assess the infection status of flies in Africa,^13^ and the collection sites in each country were selected by considering the historical information (breeding site maps, previous epidemiological data, etc.) to select productive vector breeding sites to make sure large numbers of vectors were collected.

**Figure 1.**
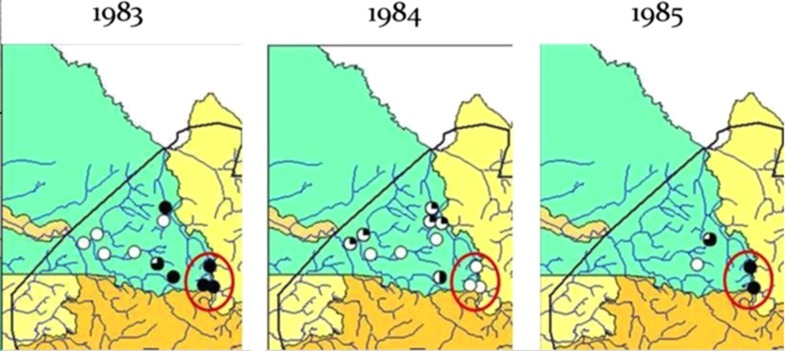
The different onchocerciasis transmission rates in Senegal from 1983 to 1985. Vector collection sites highlighted within the red circles were evaluated 3 years in a row. No infected flies were found in the second year of the collection (as indicated by the clear circles), whereas large numbers of infected flies were found in the first and third years.

Based on the recommendations of an APOC entomological working group,^14^ a minimum of six vector collection sites per focus were used, although additional sites could be added if required in order to adequately cover the transmission zone. Vector collections were done by four vector collectors catching simultaneously from 07.00–18.00 h, twice weekly, at each site during the transmission season (usually 4–5 months). The catches started at least 5 months after the last MDA with ivermectin. The entomological assessments were to be done for two consecutive years, a recommendation that was not included in the 2016 guidelines,^1^ because of concerns that seasonal variations from year-to-year could yield misleading results if assessments are done for only 1 year. An example from the work done by the Onchocerciasis Control Programme (OCP) that illustrates this variation occurred in Senegal, where inconsistent data were detected between collections performed over 3 years (Figure 1). In addition to collecting blackflies at catching sites in known hyper- and meso-endemic areas, they were collected in hypo-endemic areas. Collected flies were stored in 80% alcohol in the field and sent to the laboratory for pool screening PCR analysis using the O150 PCR.^15,16^

**Figure 2.**
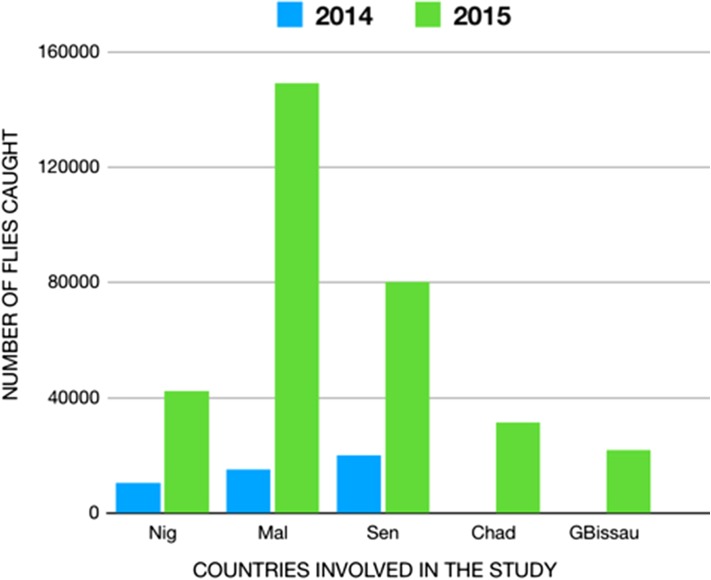
The number of flies collected in the selected countries over 2 years (2014, 2015), the clear yearly differences reflecting the need for training and better strategies. Nig=Niger, Mal=Malawi, Sen=Senegal, Chad=Chad, Gbissau=Guinea-Bissau.

Three countries performed collections in 2014 and 2015. Senegal collected 20 109 flies and 80 372 flies, respectively; Malawi collected 15 083 flies and 149 282 flies; Niger collected 10 405 flies and 42 414 flies. Two countries, Chad and Guinea Bissau, collected flies only in 2015. Chad collected 31 627 flies. Unfortunately, the collections started late in the transmission season and collections occurred in only eight out of 15 of the selection sites. In Guinea Bissau, 31 627 flies were captured. The number of flies caught in each country increased fourfold or more during the second year of collection. Analysis of the 2-year collections for all five countries has not been completed, so the results presented here should be considered preliminary (Figure 2).

## Findings

### 

Onchocerciasis is endemic in southwestern Niger on the western side of the River Niger, a region that shares borders with Burkina Faso, Benin and Nigeria. This area formed the north-easternmost part of the old OCP zone. A decade of transmission assessment data from the OCP (1981–1991) demonstrated an annual transmission potential (ATP) consistently below the lower threshold of 100, which was felt to be sufficient for the elimination of morbidity. Niger was therefore, never placed under ivermectin treatment for onchocerciasis during the time of the APOC. The only ivermectin treatments administered in the onchocerciasis endemic areas were those given for lymphatic filariasis (LF); these have recently been stopped. Entomological assessments were performed throughout the OCP-defined transmission area, and can be seen in Table 1; no infective flies were identified in either year.

**Table 1.** *Onchocerca volvulus* infection status of flies: O150 positivity in infected flies collected in 2015

Country	Evaluation area	Presence of O150-positive flies*
Niger	General	–
Malawi**	MDA areas	+*
	Non-MDA areas	+*
Senegal	Faleme Basin	–
	Gambia Basin	–
Guinea Bissau	Gabu	+*
Chad	Southern areas	+*

*+Indicates positivity seen in flies from more than one location. **Four of the five sites in the border regions with Mozambique had positive flies, whereas nine other internal sites were negative. Two sites that had not been previously under MDA treatment (i.e. in all likelihood hypo-endemic regions) both carried infected flies.

### Malawi

**Figure 3.**
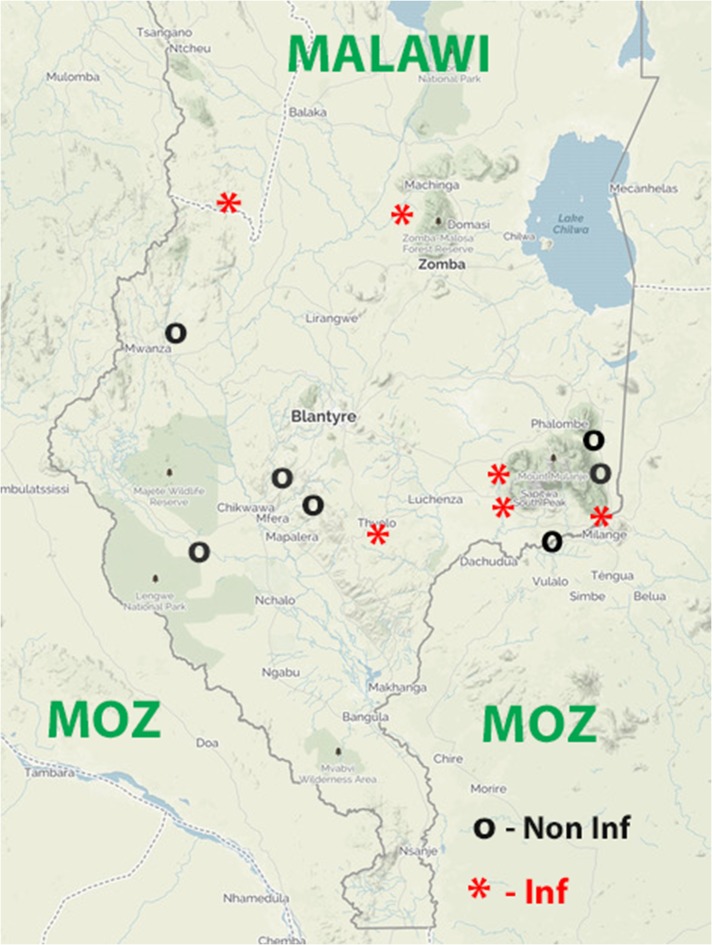
Map of Malawi showing collection sites evaluated in 2015. Collection sites where infective flies, as determined by O150 PCR, were found are indicated by yellow circles. The other collection sites, where no infective flies were found by O150 PCR, are indicated by clear circles.

The early Rapid Epidemiologic Mapping for Onchocerciasis and Rapid Epidemiological Assessment (REMO/REA) data for Malawi showed that there was onchocerciasis present in the mid-north, central and southern parts of the country. Areas of transmission were adjacent to Mozambique, Tanzania and Zambia. However, except for the southern focus, many of the border areas were thought to be hypo-endemic. Ivermectin treatment for disease control has only been administered the eight of the southern districts, beginning in three districts in 1997 and extending in 2000 to the remaining districts. Epidemiological assessments using skin-snips in 2012 indicated that all but two sites had no positive individuals.^12^ Entomological evaluations in 2014 and 2015 were carried out at all eight districts under MDA, and two adjacent districts that had been treated for LF, but not onchocerciasis, and that had highly productive *S. damnosum* breeding sites. Infective flies were found in multiple capture points in the country (Table 1). Transmission was seen both in areas under MDA and areas that had recently received MDA for LF only, but had since stopped treatment. Some of the breeding sites where infective flies were identified included border areas. All of the sites that were treated for LF only were in areas considered hypo-endemic for onchocerciasis. The breeding sites are indicated in Figure 3.

### Senegal

Onchocerciasis was defined by the OCP as being endemic in three regions of the country distributed within eight health districts. These endemic areas are within the Rivers Gambia and Falémé basins, and share borders with Guinea Bissau, Guinea and Mali. Although Senegal was included in the western extension of OCP, no vector control was ever carried out in the country. The distribution of ivermectin to endemic communities began in 1988, with a move to community-directed treatment with ivermectin (CDTI) in 1998, which was integrated with treatments for LF and schistosomiasis in 2008. The studies of Diawara et al.^4^ in 2009 and Traoré et al.^7^ in 2012, which used skin-snip microscopy to evaluate the presence of infection in humans, failed to identify infections in humans or the vector in the study area, thus prompting the need for entomological assessment over the entire endemic area. Serological evaluation will also be needed, particularly as a study found Ov16-positive children in some of the study areas.^17^ The vector collections in 2014 and 2015 were done at all endemic districts in the country; no infective flies were found (Table 1).

### Chad

The REMO/REA data showed onchocerciasis to be endemic particularly in the south of the country that shares borders with Cameroon and Central African Republic. Ivermectin treatment began in 1993, with CDTI in 1998. As the epidemiological studies using skin-snip microscopy carried out 18 years later, in 2016, suggested that the country had greatly suppressed and possibly interrupted transmission,^12^ entomological assessments were needed to confirm this suggestion. However, although vector collection sites were selected across the entire endemic area, collections were done at only eight sites due to the management and technical issues reasons related to the very short breeding season; infective flies were nevertheless found in the collections carried out in 2015 (Table 1).

### Guinea-Bissau

Two regions of the country were defined by the OCP as endemic for human onchocerciasis—Gabu where 15 health districts are endemic and Bofata with two endemic health districts. Ivermectin treatments started as part of the OCP Western Extension in 1990 with quarterly treatments until 1993. However, there have been intermittent interruptions due to the civil unrest that plagued the country. There were no treatments administered between 1998 and 2000, nor from 2002 to 2008, although annual MDA had been carried out since then. Programme evaluations suggested that the country probably had suppressed treatment (APOC, unpublished data). Fifteen collection points were identified for the black fly collection in 2015 but, due to limited in-country capacity for the collections, black flies were collected in only five of the collection points. Nonetheless, infective flies were identified, with some of the infective flies found in a collection point near the border with Guinea.

The results from this analysis of more than 400 000 *Simulium* flies have now been shared with these countries.

## Comments

This multi-country review has resulted in two important achievements:

1. The entomological data collected have provided important data on countries’ current transmission status, information that can help programs prioritize their activities (e.g. pursue serological evaluations or identify the reasons for the presence of continued transmission, etc.).
2. The review of the results has identified important issues that country programs will need to address.

The 2014 and 2015 black fly collections did not find any evidence for transmission in black flies in Niger. In order to fulfill the current criteria described in WHO guidelines for successful elimination, a priority now is to collect serological evidence to demonstrate interruption of transmission, so that the essential post-treatment surveillance phase can begin. Similarly, the results described here from Senegal are extremely encouraging and suggest that there is successful progress towards elimination. Serological evaluations are needed here as well, in order to determine if the program has met the criteria for stopping MDA.

Aside from these very encouraging results, there was convincing evidence from the PCR testing of flies caught in the other three countries that there was still ongoing transmission in some parts of these countries. Programs will need to review the data in detail to determine the most appropriate actions. However, some conclusions can be drawn from the data presented. In Malawi, for example, where considerable numbers of flies were collected and assessed, there is ongoing transmission in areas that have been treated for onchocerciasis for many years and in hypo-endemic areas that have received 5–6 years of annual ivermectin as part of MDA for LF. Some of the Malawi breeding sites were infective flies were found were along the border with Mozambique. These adjacent areas in Mozambique are areas that were defined by REMO/REA in the past as being hypo-endemic. These findings underscore the importance of understanding such hypo-endemic and cross-border areas throughout Africa. Evaluations with Ov-16 serology would greatly assist the understanding of the status of the infection in these situations and could help indicate the extent to which treatment may need to be scaled-up in areas that are hypo-endemic for onchocerciasis. In Guinea-Bissau, there was also evidence of positivity in the border areas with Guinea, again with the possibility of cross-border transmission. Despite the finding of infective flies in all three of these countries, there were areas where no infective flies were found (data not shown), so countries will need to review their site-specific data and determine where serological evaluations could be implemented as part of a stop-MDA survey and where serological evaluations could be implemented as part of routine evaluations of transmission.

The earlier reports from the APOC-supported epidemiological surveys carried out before this body closed had generated much optimism about the status of transmission of onchocerciasis before the essential entomological evaluations had been carried out. These entomological evaluations presented here do not entirely confirm this optimism, although they do not diminish the accomplishments of the programs in controlling the morbidity of the disease. It has been known for some time that skin-snip microscopy has a reduced sensitivity in areas of low transmission,^18,19^ and so it is not surprising that the entomological and epidemiological assessment came to differing conclusions. These results add to the data that support the necessity of both human and vector surveys when determining where it is acceptable to stop MDA for onchocerciasis. Assessing the human populations with Ov-16 serology will be an important additional step that will provide essential information needed to make the major program decisions, such as stopping MDA treatment or changing treatment strategies.

There were major differences between the numbers of flies collected between 2014 and 2015. Factors that may have contributed to the low catches in the first year of collection in at least some countries included limited capacity (in terms of both number of collectors and training of skill sets of collectors), a need for better timing of the actual collections, and annual variations in fly populations due to variable rain patterns. Attempts were made to address the different program factors after the 2014 activities, and this could explain the improved collections seen in 2015. Programs should invest the time to “ground truth” their vector collection sites by determining the current seasonal changes in fly populations and verifying the productivity of the various sites. Additionally, programs should take into account the possibility of a reduced fly population in some years due to abnormal rain patterns. Low collections could necessitate the calculation of the ATP or an additional year of collection. In either case, continued investments will be needed to ensure appropriate preparatory activities for entomological collections and to ensure the capacity to analyze the black flies in a timely manner. In this latter context, it is must be recognized that, as the different endemic countries move closer to onchocerciasis elimination, there is likely to be a large increase in the number of flies needing appropriate analysis. It is important that suitable laboratory facilities and support are available to accommodate this need.

## Conclusions

Although earlier epidemiological surveys using skin-snips had indicated that MDA could probably be stopped countrywide in some instances, this conclusion was not supported by entomological assessment. Nevertheless, the entomological assessments identified two countries that should be prioritized for serological evaluation to determine if MDA is needed. Although the entomological data from the other three countries demonstrated ongoing transmission in at least some areas, it is possible that there may be foci of transmission where a serological evaluation in children could demonstrate interruption of transmission. The final determination about this issue will require detailed review of the data by the country programs. In any case, these data add support to the continually expanding body of evidence that onchocerciasis can, indeed, be eliminated from Africa.^5^

Several key challenges to the elimination programs were identified. First, there is a need for better training and increased program expertise in the entomological evaluation, with a particular need to understand the seasonality and productivity of breeding sites. Secondly, it is clear that areas defined as hypo-endemic need evaluation to determine whether transmission is occurring in these areas. In Malawi, such transmission was found even after 5 years of ivermectin and albendazole MDA for LF. Finally, there is an urgent need to encourage country-to-country collaboration in order to address potential cross-border transmission of onchocerciasis.
